# Crystal structures of 4-chloro­pyridine-2-carbo­nitrile and 6-chloro­pyridine-2-carbo­nitrile exhibit different inter­molecular π-stacking, C—H⋯N_nitrile_ and C—H⋯N_pyridine_ inter­actions

**DOI:** 10.1107/S2056989015011767

**Published:** 2015-06-27

**Authors:** Matthew J. Montgomery, Thomas J. O’Connor, Joseph M. Tanski

**Affiliations:** a Department of Chemistry, Vassar College, Poughkeepsie, NY 12604, USA

**Keywords:** crystal structure, chloro­cyano­pyridine, π-stacking, C—H⋯N inter­actions

## Abstract

The two title compounds are isomers of C_6_H_3_ClN_2_ containing a pyridine ring, a nitrile group, and a chloro substituent. The mol­ecules of each compound pack together in the solid state with offset face-to-face π-stacking, and inter­molecular C—H⋯N_nitrile_ and C—H⋯N_pyridine_ inter­actions. 4-Chloro­pyridine-2-carbo­nitrile, (I), exhibits pairwise centrosymmetric head-to-head C—H⋯N_nitrile_ and C—H⋯N_pyridine_ inter­actions, forming one-dimensional chains, which are π-stacked in an offset face-to-face fashion. The inter­molecular packing of the isomeric 6-chloro­pyridine-2-carbo­nitrile, (II), which differs only in the position of the chloro substituent on the pyridine ring, exhibits head-to-tail C—H⋯N_nitrile_ and C—H⋯N_pyridine_ inter­actions, forming two-dimensional sheets which are π-stacked in an offset face-to-face fashion. In contrast to (I), the offset face-to-face π-stacking in (II) is formed between mol­ecules with alternating orientations of the chloro and nitrile substituents.

## Abstract 2

The crystal structures of two chloro­cyano­pyridines, namely 4-chloro­pyridine-2-carbo­nitrile and 6-chloro­pyridine-2-carbo­nitrile, exhibit unique inter­molecular C—H⋯N_nitrile_, C—H⋯N_pyridine_ and offset face-to-face π-stacking inter­actions.

## Chemical context   

Chloro­pyridine­carbo­nitriles are members of a class of compounds containing the ubiquitous six-membered nitro­gen-containing heterocycle pyridine. The pyridine heterocycle features prominently in many valuable synthetic compounds (Bull *et al.*, 2012 ▸). While several of the ten possible isomers of chloro­pyridine­carbo­nitrile are commercially available, none of their crystal structures have been reported in the literature, although the structure of 2-chloro­pyridine-4-carbo­nitrile has been deposited in the Cambridge Structural Database (Version 5.31, June 2015 with updates; Groom & Allen, 2014 ▸) as a private communication (refcode LOBVIJ). The title compounds represent two isomers of chloro­pyridine-2-carbo­nitrile, namely 4-chloro­pyridine-2-carbo­nitrile, (I), and 6-chloro­pyridine-2-carbo­nitrile, (II). In both cases, the intra­molecular packing exhibits weak inter­molecular C—H⋯N inter­actions, which are well documented (Desiraju & Steiner, 1999 ▸), as well as aromatic π-stacking inter­actions (Hunter & Saunders, 1990 ▸; Lueckheide *et al.*, 2013 ▸).
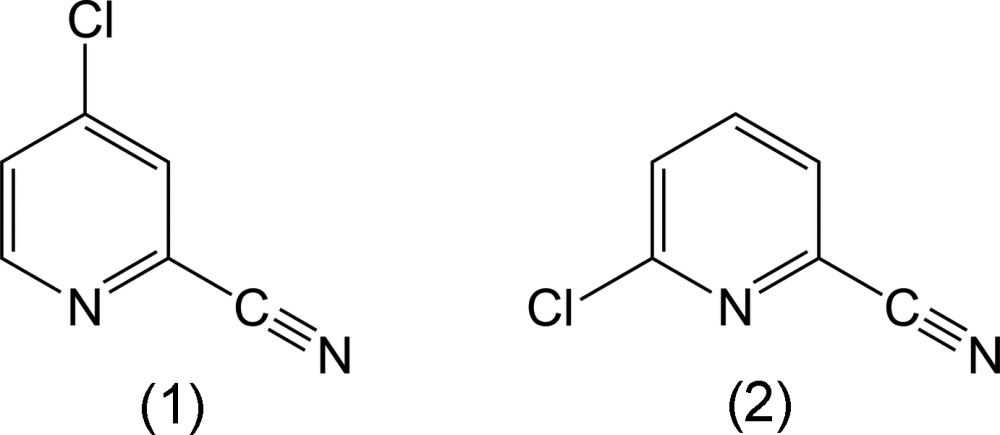


4-Chloro­pyridine-2-carbo­nitrile, (I), may be synthesized by the cyanation of 4-chloro­pyridine *N*-oxide with tri­methyl­silanecarbo­nitrile (TMSCN) (Sakamoto *et al.*, 1985 ▸). More recently, it has been shown that (I) can be prepared in a one-step process from 4-nitro­pyridine *N*-oxide with ethyl chloro­formate and TMSCN (Veerareddy *et al.*, 2011 ▸). (I) has found use as a building block for a family of chiral catalysts (Busto *et al.*, 2005 ▸).

6-Chloro­pyridine-2-carbo­nitrile, (II), may be synthesized by the vapor-phase chlorination of 2-cyano­pyridine (Ruetman & Taplin, 1971 ▸), or by the cyanation of 2-chloro­pyridine *N*-oxide hydro­chloride with sodium cyanide (Tsukamoto *et al.*, 2009 ▸). This compound has found applications in the preparation of biologically active or pharmaceutical compounds, such as heteroaromatic carb­oxy­lic acids (Kiener *et al.*, 1996 ▸) and 2-aryl­amino-substituted pyridinyl nitriles (Guo *et al.*, 2013 ▸).

## Structural commentary   

**Figure 1:**
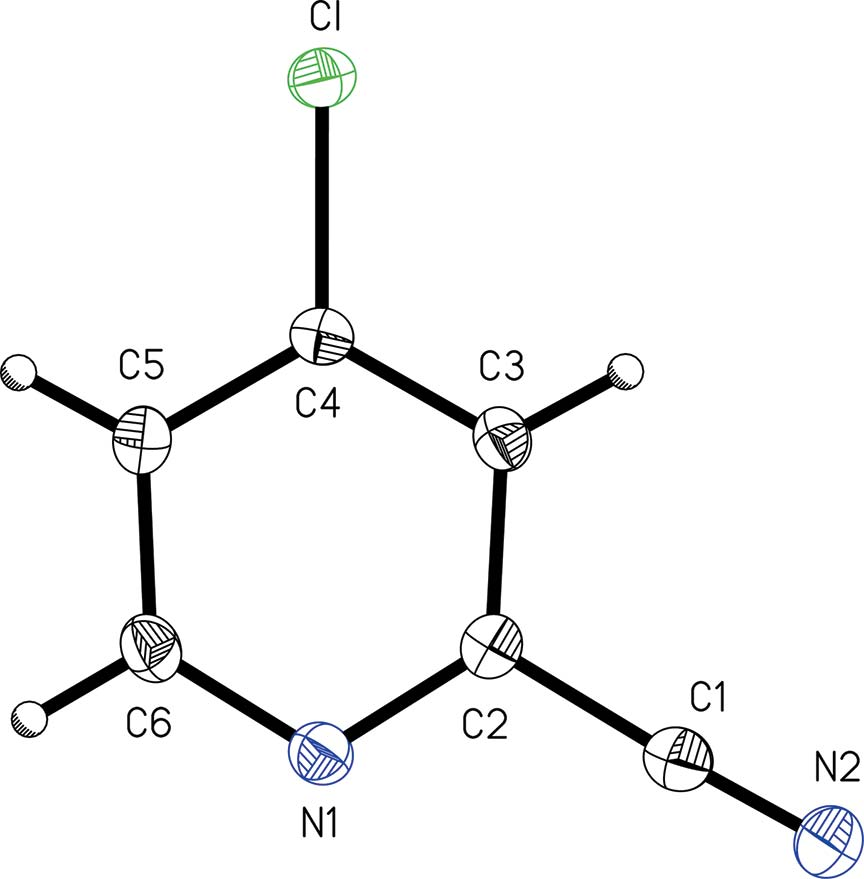
A view of 4-chloro­pyridine-2-carbo­nitrile, (I), with the atom-numbering scheme. Displacement ellipsoids are shown at the 50% probability level.

**Figure 2:**
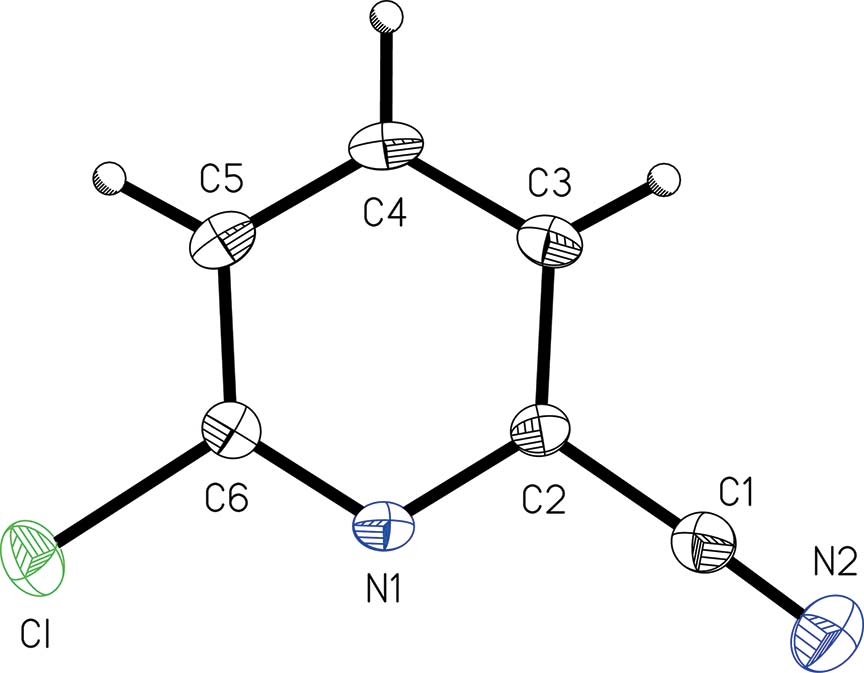
A view of 6-chloro­pyridine-2-carbo­nitrile, (II), with the atom-numbering scheme. Displacement ellipsoids are shown at the 50% probability level.

4-Chloro­pyridine-2-carbo­nitrile, (I) (Fig. 1 ▸), and 6-chloro­pyridine-2-carbo­nitrile, (II) (Fig. 2 ▸), exhibit similar metrical parameters. The nitrile bond length C1—N2 of 1.156 (3) Å in (I) and 1.138 (2) Å in (II) are similar to those seen in the related structure 2-chloro­pyridine-4-carbo­nitrile, with the nitrile C≡N distance is 1.141 Å (CSD refcode LOBVIJ). The nitrile bond lengths in 2- and 3-cyano­pyridine [1.145 (2) and 1.150 (1) Å, respectively; Kubiak *et al.*, 2002 ▸] and 4-cyano­pyridine [1.137 (8) Å; Laing *et al.*, 1971 ▸] are also similar to those found in the title compounds. The aromatic chlorine bond lengths, *viz.* C4—Cl and C6—Cl of 1.740 (3) Å in (I) and 1.740 (1) Å in (II), are similar to those seen in the related structures 2-chloro­pyridine-4-carbo­nitrile (1.732 Å; CSD refcode LOBVIJ), 2- and 3-chloro­pyridine hydro­chloride (1.710 and 1.727 Å, respectively; Freytag & Jones, 2001 ▸), and 4-chloro­pyridine hydro­chloride (1.730 Å; Freytag *et al.*, 1999 ▸).

Both (I) and (II) are almost planar, with r.m.s. deviations from the mean planes of all non-H atoms of 0.0077 and 0.0161 Å, respectively. As may be expected, the heterocyclic rings are slightly wedge shaped as the pyridine C—N bond are shorter than the C—C bonds in each aromatic ring. In (I), the ring C2—N1 and C6—N1 bond lengths of 1.361 (3) and 1.350 (3) Å are similar to those found in (II) of 1.349 (1) and 1.322 (1) Å. The average ring C—C bond lengths are 1.403 (2) Å in (I) and 1.391 (5) Å in (II). The lengths are comparable to those found in the parent compound, pyridine, with C—N of 1.34 Å and C—C of 1.38 Å (Mootz & Wussow, 1981 ▸), and in the related structure 2-chloro­pyridine-4-carbo­nitrile, with C—N bond lengths of 1.328 and 1.340 Å, and an average C—C bond length of 1.377 (7) Å (CSD refcode LOBVIJ).

## Supra­molecular features   

The mol­ecules of each of the title compounds pack together in the solid state with π-stacking, and inter­molecular C—H⋯N_nitrile_ and C—H⋯N_pyridine_ inter­actions, however, the packing motifs are unique, and also different than those found in the related structure 2-chloro­pyridine-4-carbo­nitrile (CSD refcode LOBVIJ). For a discussion of weak C—H⋯*X* inter­actions, see Desiraju & Steiner (1999 ▸).

**Figure 3:**
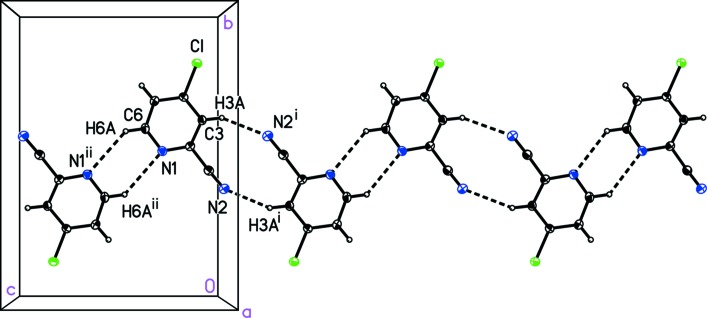
A view of the inter­molecular C—H⋯N_nitrile_ and C—H⋯N_pyridine_ contacts (dashed lines) in 4-chloro­pyridine-2-carbo­nitrile, (I), that form a one-dimensional chain. [Symmetry codes: (i) −*x* − 1, −*y* + 1, −*z*; (ii) −*x*, −*y* + 1, −*z* + 1.]

**Figure 4:**
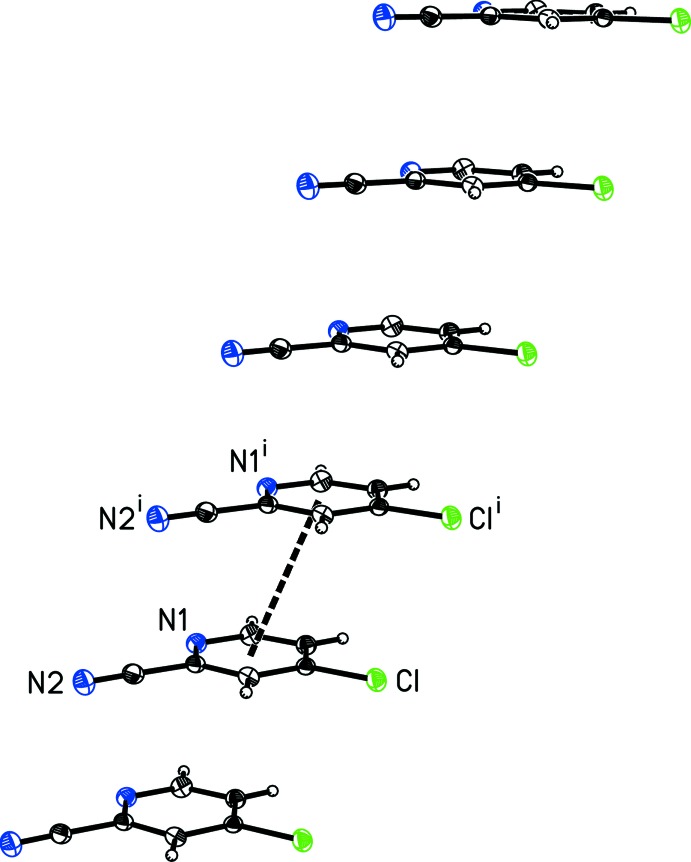
A view of the offset face-to-face π-stacking in 4-chloro­pyridine-2-carbo­nitrile, (I), with the thick dashed line indicating a centroid-to-centroid inter­action. [Symmetry code: (i) *x* + 1, *y*, *z*.]

The mol­ecules of (I) pack together in the solid state *via* alternating centrosymmetric head-to-head inter­molecular C—H⋯N_nitrile_ and C—H⋯N_pyridine_ inter­actions to form a one-dimensional zigzag chain (Fig. 3 ▸ and Table 1 ▸). The chains further pack together through offset face-to-face π-stacking (Fig. 4 ▸). This π-stacking is characterized by a centroid-to-centroid distance of 3.813 (5) Å, a plane-to-centroid distance of 3.454 (4) Å, and a ring offset or ring-slippage distance of 1.615 (3) Å (Hunter & Saunders, 1990 ▸; Lueckheide *et al.*, 2013 ▸). The π-stacking in (I) is similar to that found in the related unpublished structure 2-chloro­pyridine-4-carbo­nitrile (CSD refcode LOBVIJ).

**Figure 5:**
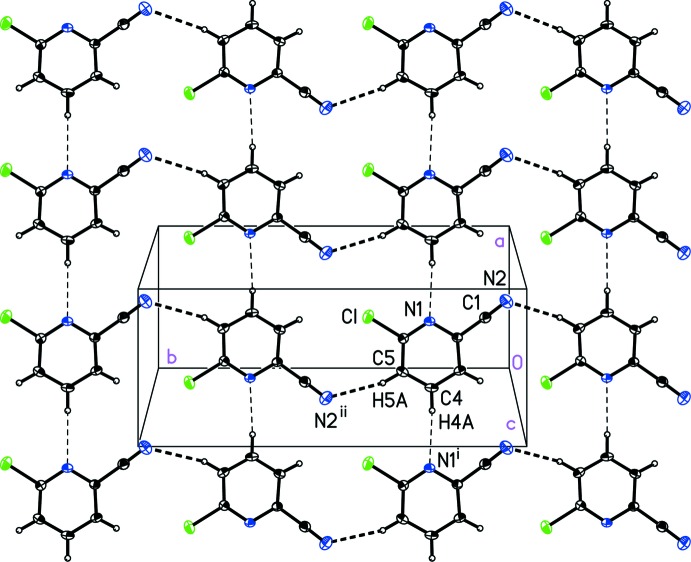
A view of the inter­molecular C—H⋯N_nitrile_ and C—H⋯N_pyridine_ contacts (dashed lines) in 6-chloro­pyridine-2-carbo­nitrile, (I), that form a two-dimensional sheet. [Symmetry codes: (i) *x* − 1, *y*, *z*; (ii) −*x* + 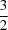, *y* − 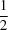, −*z* + 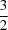.]

**Figure 6:**
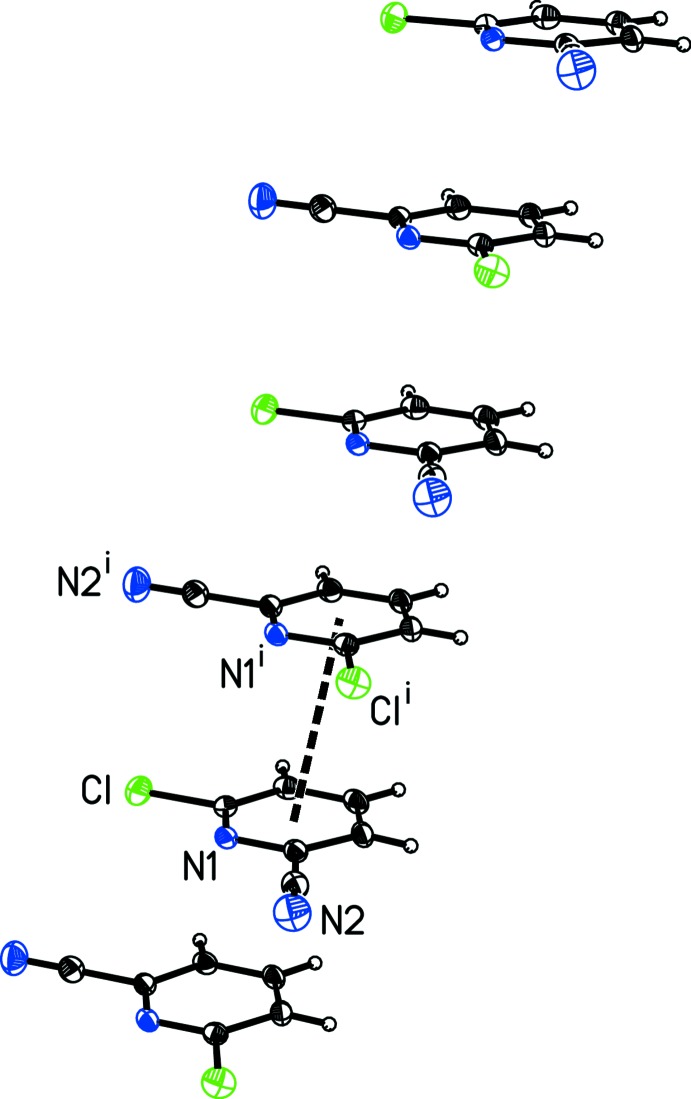
A view of the alternating offset face-to-face π-stacking in 6-chloro­pyridine-2-carbo­nitrile, (II), with the thick dashed line indicating a centroid-to-centroid inter­action. [Symmetry code: (i) *x* + 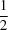, −*y* + 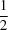, *z* + 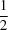.]

In contrast to (I), the mol­ecules of (II) pack together *via* head-to-tail C—H⋯N_nitrile_ and C—H⋯N_pyridine_ inter­actions to form two-dimensional sheets that are parallel to the (001) plane (Fig. 5 ▸ and Table 2 ▸). As in (I), the parallel planes of the mol­ecules engage in offset face-to-face π-stacking between the two-dimensional sheets, which is characterized by a ring centroid-to-centroid distance of 3.7204 (7) Å, a centroid-to-plane distance of 3.41 (1) Å, and a ring-offset slippage of 1.48 (2) Å (Fig. 6 ▸). However, in constrast to (I), the π-stacking in (II) is formed between mol­ecules with alternating orientations of the chloro and nitrile substituents with a plane-to-plane angle of 0.23 (5)°. For a more thorough description of π-stacking, see Hunter & Saunders (1990 ▸) and Lueckheide *et al.* (2013 ▸).

Notably, there are no significant Cl⋯Cl contacts in (I) or (II), in contrast to 2-chloro­pyridine-4-carbo­nitrile (CSD refcode LOBVIJ), which exhibits a Cl⋯Cl contact distance of 3.371 Å that is shorter than the sum of the van der Waals radius of chlorine (3.5 Å; Bondi, 1964 ▸). For more information on halide–halide contacts, see Pedireddi *et al.* (1994 ▸) and Jelsch *et al.* (2015 ▸).

## Synthesis and crystallization   

4-Chloro­pyridine-2-carbo­nitrile (97%) and 6-chloro­pyridine-2-carbo­nitrile (96%) were purchased from Aldrich Chemical Company, USA. 4-Chloro­pyridine-2-carbo­nitrile was recrystallized from 95% ethanol.

## Refinement   

Crystal data, data collection and structure refinement details are summarized in Table 3 ▸. H atoms on C atoms were included in calculated positions and refined using a riding model, with C—H = 0.95 Å and *U*_iso_(H) = 1.2*U*_eq_(C) of the aryl C atoms.

## Supplementary Material

Crystal structure: contains datablock(s) global, I, II. DOI: 10.1107/S2056989015011767/rz5161sup1.cif

Structure factors: contains datablock(s) I. DOI: 10.1107/S2056989015011767/rz5161Isup2.hkl

Structure factors: contains datablock(s) II. DOI: 10.1107/S2056989015011767/rz5161IIsup3.hkl

Supporting information file. DOI: 10.1107/S2056989015011767/rz5161Isup4.cml

Supporting information file. DOI: 10.1107/S2056989015011767/rz5161IIsup5.cml

CCDC references: 1407613, 1407612

Additional supporting information:  crystallographic information; 3D view; checkCIF report

## Figures and Tables

**Table 1:** Hydrogen-bond geometry (Å, °) for (I)

*D*—H⋯*A*	*D*—H	H⋯*A*	*D*⋯*A*	*D*—H⋯*A*
C3—H3*A*⋯N2^i^	0.95	2.64	3.462 (5)	146
C6—H6*A*⋯N1^ii^	0.95	2.75	3.493 (5)	136

Symmetry codes: (i) 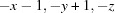; (ii) 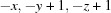.

**Table 2:** Hydrogen-bond geometry (Å, °) for (II)

*D*—H⋯*A*	*D*—H	H⋯*A*	*D*⋯*A*	*D*—H⋯*A*
C4—H4*A*⋯N1^i^	0.95	2.49	3.4099 (15)	164
C5—H5*A*⋯N2^ii^	0.95	2.70	3.5651 (17)	152

Symmetry codes: (i) 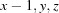; (ii) 
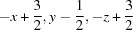
.

**Table 3:** Experimental details

	(I)	(II)
Crystal data		
Chemical formula	C_6_H_3_ClN_2_	C_6_H_3_ClN_2_
*M*_r_	138.55	138.55
Crystal system, space group	Monoclinic, *P*2_1_/*n*	Monoclinic, *P*2_1_/*n*
Temperature (K)	125	125
*a*, *b*, *c* (Å)	3.813 (5), 14.047 (19), 11.356 (15)	6.1739 (15), 15.238 (4), 7.0123 (18)
β (°)	96.806 (19)	112.492 (4)
*V* (Å^3^)	604.0 (14)	609.5 (3)
*Z*	4	4
Radiation type	Mo *K*α	Mo *K*α
μ (mm^−1^)	0.52	0.52
Crystal size (mm)	0.25 × 0.10 × 0.04	0.20 × 0.15 × 0.03

Data collection		
Diffractometer	Bruker APEXII CCD	Bruker APEXII CCD
Absorption correction	Multi-scan (*SADABS*; Bruker, 2013 ▸)	Multi-scan (*SADABS*; Bruker, 2013 ▸)
*T*_min_, *T*_max_	0.67, 0.98	0.82, 0.98
No. of measured, independent and observed [*I* > 2σ(*I*)] reflections	12191, 1852, 1498	15460, 1868, 1657
*R*_int_	0.063	0.031
(sin θ/λ)_max_ (Å^−1^)	0.715	0.717

Refinement		
*R*[*F* ^2^ > 2σ(*F* ^2^)], *wR*(*F* ^2^), *S*	0.050, 0.135, 1.12	0.028, 0.082, 1.09
No. of reflections	1852	1868
No. of parameters	82	82
H-atom treatment	H-atom parameters constrained	H-atom parameters constrained
Δρ_max_, Δρ_min_ (e Å^−3^)	0.53, −0.37	0.48, −0.19

Computer programs: *APEX2* and *SAINT* (Bruker, 2013 ▸), *SHELXS2014* and *SHELXTL2014* (Sheldrick, 2008 ▸), *SHELXL2014* (Sheldrick, 2015 ▸), *OLEX2* (Dolomanov *et al.*, 2009 ▸) and *Mercury* (Macrae *et al.*, 2008 ▸).

## supplementary crystallographic information

### (I) 4-Chloropyridine-2-carbonitrile Crystal data

**Table:**

C_6_H_3_ClN_2_	*F*(000) = 280
*M**_r_* = 138.55	*D*_x_ = 1.524 Mg m^−^^3^
Monoclinic, *P*2_1_/*n*	Mo *K*α radiation, λ = 0.71073 Å
*a* = 3.813 (5) Å	Cell parameters from 3637 reflections
*b* = 14.047 (19) Å	θ = 2.9–30.3°
*c* = 11.356 (15) Å	µ = 0.52 mm^−^^1^
β = 96.806 (19)°	*T* = 125 K
*V* = 604.0 (14) Å^3^	Plate, colourless
*Z* = 4	0.25 × 0.10 × 0.04 mm

### (I) 4-Chloropyridine-2-carbonitrile Data collection

**Table:**

Bruker APEXII CCD diffractometer	1852 independent reflections
Radiation source: fine-focus sealed tube	1498 reflections with *I* > 2σ(*I*)
Graphite monochromator	*R*_int_ = 0.063
Detector resolution: 8.3333 pixels mm^-1^	θ_max_ = 30.6°, θ_min_ = 2.3°
φ and ω scans	*h* = −5→5
Absorption correction: multi-scan (*SADABS*; Bruker, 2013)	*k* = −20→19
*T*_min_ = 0.67, *T*_max_ = 0.98	*l* = −16→16
12191 measured reflections	

### (I) 4-Chloropyridine-2-carbonitrile Refinement

**Table:**

Refinement on *F*^2^	0 restraints
Least-squares matrix: full	Hydrogen site location: inferred from neighbouring sites
*R*[*F*^2^ > 2σ(*F*^2^)] = 0.050	H-atom parameters constrained
*wR*(*F*^2^) = 0.135	*w* = 1/[σ^2^(*F*_o_^2^) + (0.0646*P*)^2^ + 0.4268*P*] where *P* = (*F*_o_^2^ + 2*F*_c_^2^)/3
*S* = 1.12	(Δ/σ)_max_ < 0.001
1852 reflections	Δρ_max_ = 0.53 e Å^−^^3^
82 parameters	Δρ_min_ = −0.37 e Å^−^^3^

### (I) 4-Chloropyridine-2-carbonitrile Special details

**Table:**

Geometry. All e.s.d.'s (except the e.s.d. in the dihedral angle between two l.s. planes) are estimated using the full covariance matrix. The cell e.s.d.'s are taken into account individually in the estimation of e.s.d.'s in distances, angles and torsion angles; correlations between e.s.d.'s in cell parameters are only used when they are defined by crystal symmetry. An approximate (isotropic) treatment of cell e.s.d.'s is used for estimating e.s.d.'s involving l.s. planes.

### (I) 4-Chloropyridine-2-carbonitrile Fractional atomic coordinates and isotropic or equivalent isotropic displacement parameters (Å^2^)

**Table:**

	*x*	*y*	*z*	*U*_iso_*/*U*_eq_	
Cl	0.27576 (14)	0.79489 (4)	0.15624 (5)	0.02207 (17)	
N1	0.0055 (5)	0.52050 (12)	0.33977 (16)	0.0197 (4)	
N2	−0.4067 (5)	0.39592 (13)	0.09730 (18)	0.0253 (4)	
C1	−0.2642 (5)	0.45592 (15)	0.15388 (19)	0.0204 (4)	
C2	−0.0811 (5)	0.53460 (14)	0.22134 (18)	0.0173 (4)	
C3	−0.0084 (5)	0.61731 (14)	0.15971 (18)	0.0177 (4)	
H3A	−0.0758	0.6235	0.0768	0.021*	
C4	0.1688 (5)	0.69050 (13)	0.22646 (18)	0.0163 (4)	
C5	0.2634 (5)	0.67930 (15)	0.34888 (18)	0.0192 (4)	
H5A	0.383	0.7283	0.3953	0.023*	
C6	0.1748 (6)	0.59298 (15)	0.40037 (19)	0.0207 (4)	
H6A	0.2382	0.5851	0.4832	0.025*	

### (I) 4-Chloropyridine-2-carbonitrile Atomic displacement parameters (Å^2^)

**Table:**

	*U*^11^	*U*^22^	*U*^33^	*U*^12^	*U*^13^	*U*^23^
Cl	0.0217 (3)	0.0212 (3)	0.0227 (3)	−0.00411 (18)	0.00040 (18)	0.00357 (18)
N1	0.0200 (8)	0.0197 (8)	0.0191 (9)	0.0004 (6)	0.0016 (7)	0.0014 (7)
N2	0.0261 (9)	0.0245 (9)	0.0247 (10)	−0.0046 (7)	0.0003 (8)	−0.0011 (7)
C1	0.0176 (9)	0.0218 (9)	0.0216 (10)	0.0002 (7)	0.0019 (8)	0.0027 (8)
C2	0.0141 (8)	0.0180 (9)	0.0199 (10)	0.0012 (7)	0.0021 (7)	−0.0016 (7)
C3	0.0161 (9)	0.0217 (9)	0.0153 (9)	−0.0004 (7)	0.0020 (7)	−0.0001 (7)
C4	0.0144 (8)	0.0169 (8)	0.0180 (9)	0.0012 (7)	0.0032 (7)	0.0018 (7)
C5	0.0185 (9)	0.0201 (9)	0.0185 (10)	0.0003 (7)	0.0002 (8)	−0.0024 (7)
C6	0.0236 (10)	0.0230 (10)	0.0152 (9)	0.0012 (8)	0.0020 (8)	−0.0004 (7)

### (I) 4-Chloropyridine-2-carbonitrile Geometric parameters (Å, º)

**Table:**

Cl—C4	1.740 (3)	C3—C4	1.402 (3)
N1—C6	1.350 (3)	C3—H3A	0.95
N1—C2	1.361 (3)	C4—C5	1.403 (3)
N2—C1	1.156 (3)	C5—C6	1.405 (3)
C1—C2	1.473 (3)	C5—H5A	0.95
C2—C3	1.401 (3)	C6—H6A	0.95
			
C6—N1—C2	116.07 (19)	C3—C4—Cl	119.61 (18)
N2—C1—C2	177.6 (2)	C5—C4—Cl	120.11 (16)
N1—C2—C3	125.12 (19)	C4—C5—C6	117.58 (19)
N1—C2—C1	116.70 (19)	C4—C5—H5A	121.2
C3—C2—C1	118.2 (2)	C6—C5—H5A	121.2
C2—C3—C4	116.7 (2)	N1—C6—C5	124.3 (2)
C2—C3—H3A	121.7	N1—C6—H6A	117.9
C4—C3—H3A	121.7	C5—C6—H6A	117.9
C3—C4—C5	120.27 (19)		
			
C6—N1—C2—C3	−0.1 (3)	C2—C3—C4—Cl	178.93 (15)
C6—N1—C2—C1	179.66 (19)	C3—C4—C5—C6	0.1 (3)
N1—C2—C3—C4	0.3 (3)	Cl—C4—C5—C6	−179.07 (16)
C1—C2—C3—C4	−179.48 (18)	C2—N1—C6—C5	−0.1 (3)
C2—C3—C4—C5	−0.3 (3)	C4—C5—C6—N1	0.1 (3)

### (I) 4-Chloropyridine-2-carbonitrile Hydrogen-bond geometry (Å, º)

**Table:**

*D*—H···*A*	*D*—H	H···*A*	*D*···*A*	*D*—H···*A*
C3—H3*A*···N2^i^	0.95	2.64	3.462 (5)	146
C6—H6*A*···N1^ii^	0.95	2.75	3.493 (5)	136

Symmetry codes: (i) −*x*−1, −*y*+1, −*z*; (ii) −*x*, −*y*+1, −*z*+1.

### (II) 6-Chloropyridine-2-carbonitrile Crystal data

**Table:**

C_6_H_3_ClN_2_	*F*(000) = 280
*M**_r_* = 138.55	*D*_x_ = 1.510 Mg m^−^^3^
Monoclinic, *P*2_1_/*n*	Mo *K*α radiation, λ = 0.71073 Å
*a* = 6.1739 (15) Å	Cell parameters from 9960 reflections
*b* = 15.238 (4) Å	θ = 2.7–30.5°
*c* = 7.0123 (18) Å	µ = 0.52 mm^−^^1^
β = 112.492 (4)°	*T* = 125 K
*V* = 609.5 (3) Å^3^	Plate, colourless
*Z* = 4	0.20 × 0.15 × 0.03 mm

### (II) 6-Chloropyridine-2-carbonitrile Data collection

**Table:**

Bruker APEXII CCD diffractometer	1868 independent reflections
Radiation source: fine-focus sealed tube	1657 reflections with *I* > 2σ(*I*)
Graphite monochromator	*R*_int_ = 0.031
Detector resolution: 8.3333 pixels mm^-1^	θ_max_ = 30.6°, θ_min_ = 2.7°
φ and ω scans	*h* = −8→8
Absorption correction: multi-scan (*SADABS*; Bruker, 2013)	*k* = −21→21
*T*_min_ = 0.82, *T*_max_ = 0.98	*l* = −10→9
15460 measured reflections	

### (II) 6-Chloropyridine-2-carbonitrile Refinement

**Table:**

Refinement on *F*^2^	0 restraints
Least-squares matrix: full	Hydrogen site location: inferred from neighbouring sites
*R*[*F*^2^ > 2σ(*F*^2^)] = 0.028	H-atom parameters constrained
*wR*(*F*^2^) = 0.082	*w* = 1/[σ^2^(*F*_o_^2^) + (0.0424*P*)^2^ + 0.1697*P*] where *P* = (*F*_o_^2^ + 2*F*_c_^2^)/3
*S* = 1.09	(Δ/σ)_max_ = 0.001
1868 reflections	Δρ_max_ = 0.48 e Å^−^^3^
82 parameters	Δρ_min_ = −0.19 e Å^−^^3^

### (II) 6-Chloropyridine-2-carbonitrile Special details

**Table:**

Geometry. All e.s.d.'s (except the e.s.d. in the dihedral angle between two l.s. planes) are estimated using the full covariance matrix. The cell e.s.d.'s are taken into account individually in the estimation of e.s.d.'s in distances, angles and torsion angles; correlations between e.s.d.'s in cell parameters are only used when they are defined by crystal symmetry. An approximate (isotropic) treatment of cell e.s.d.'s is used for estimating e.s.d.'s involving l.s. planes.

### (II) 6-Chloropyridine-2-carbonitrile Fractional atomic coordinates and isotropic or equivalent isotropic displacement parameters (Å^2^)

**Table:**

	*x*	*y*	*z*	*U*_iso_*/*U*_eq_	
Cl	1.01050 (5)	0.09596 (2)	0.82065 (4)	0.02803 (10)	
N1	0.98012 (14)	0.26545 (6)	0.82258 (13)	0.01720 (17)	
N2	1.10287 (19)	0.47889 (7)	0.79286 (18)	0.0351 (2)	
C1	1.00059 (18)	0.41966 (8)	0.81170 (17)	0.0232 (2)	
C2	0.87412 (16)	0.34169 (7)	0.83298 (15)	0.01728 (19)	
C3	0.66223 (17)	0.34787 (7)	0.85770 (15)	0.0198 (2)	
H3A	0.5958	0.4032	0.8664	0.024*	
C4	0.55044 (17)	0.26976 (8)	0.86928 (16)	0.0210 (2)	
H4A	0.4041	0.271	0.8846	0.025*	
C5	0.65405 (17)	0.19009 (7)	0.85828 (15)	0.0205 (2)	
H5A	0.5818	0.1359	0.8659	0.025*	
C6	0.86897 (17)	0.19295 (7)	0.83550 (15)	0.01759 (19)	

### (II) 6-Chloropyridine-2-carbonitrile Atomic displacement parameters (Å^2^)

**Table:**

	*U*^11^	*U*^22^	*U*^33^	*U*^12^	*U*^13^	*U*^23^
Cl	0.03124 (16)	0.02096 (15)	0.03084 (16)	0.00422 (9)	0.01070 (11)	−0.00400 (9)
N1	0.0135 (3)	0.0212 (4)	0.0168 (4)	−0.0004 (3)	0.0058 (3)	−0.0014 (3)
N2	0.0343 (5)	0.0294 (5)	0.0430 (6)	−0.0068 (4)	0.0164 (5)	0.0013 (4)
C1	0.0207 (5)	0.0237 (5)	0.0250 (5)	0.0006 (4)	0.0085 (4)	−0.0006 (4)
C2	0.0148 (4)	0.0199 (4)	0.0167 (4)	−0.0007 (3)	0.0055 (3)	−0.0001 (3)
C3	0.0154 (4)	0.0238 (5)	0.0204 (4)	0.0035 (3)	0.0071 (3)	0.0010 (4)
C4	0.0135 (4)	0.0317 (5)	0.0187 (4)	−0.0012 (4)	0.0072 (3)	0.0010 (4)
C5	0.0182 (4)	0.0246 (5)	0.0186 (4)	−0.0051 (3)	0.0068 (3)	0.0004 (4)
C6	0.0175 (4)	0.0193 (4)	0.0151 (4)	0.0004 (3)	0.0052 (3)	−0.0012 (3)

### (II) 6-Chloropyridine-2-carbonitrile Geometric parameters (Å, º)

**Table:**

Cl—C6	1.7402 (11)	C3—C4	1.3938 (15)
N1—C6	1.3218 (13)	C3—H3A	0.95
N1—C2	1.3490 (13)	C4—C5	1.3881 (16)
N2—C1	1.1378 (16)	C4—H4A	0.95
C1—C2	1.4604 (15)	C5—C6	1.3965 (14)
C2—C3	1.3870 (14)	C5—H5A	0.95
			
C6—N1—C2	116.15 (9)	C5—C4—H4A	120.2
N2—C1—C2	177.99 (12)	C3—C4—H4A	120.2
N1—C2—C3	124.44 (9)	C4—C5—C6	117.21 (9)
N1—C2—C1	113.94 (9)	C4—C5—H5A	121.4
C3—C2—C1	121.62 (9)	C6—C5—H5A	121.4
C2—C3—C4	117.46 (9)	N1—C6—C5	125.09 (9)
C2—C3—H3A	121.3	N1—C6—Cl	114.83 (8)
C4—C3—H3A	121.3	C5—C6—Cl	120.07 (8)
C5—C4—C3	119.65 (9)		
			
C6—N1—C2—C3	0.62 (14)	C3—C4—C5—C6	−0.07 (15)
C6—N1—C2—C1	−178.24 (8)	C2—N1—C6—C5	0.12 (15)
N1—C2—C3—C4	−1.03 (15)	C2—N1—C6—Cl	179.80 (7)
C1—C2—C3—C4	177.74 (9)	C4—C5—C6—N1	−0.38 (15)
C2—C3—C4—C5	0.71 (15)	C4—C5—C6—Cl	179.96 (7)

### (II) 6-Chloropyridine-2-carbonitrile Hydrogen-bond geometry (Å, º)

**Table:**

*D*—H···*A*	*D*—H	H···*A*	*D*···*A*	*D*—H···*A*
C4—H4*A*···N1^i^	0.95	2.49	3.4099 (15)	164
C5—H5*A*···N2^ii^	0.95	2.70	3.5651 (17)	152

Symmetry codes: (i) *x*−1, *y*, *z*; (ii) −*x*+3/2, *y*−1/2, −*z*+3/2.
